# ARGOS8 variants generated by CRISPR‐Cas9 improve maize grain yield under field drought stress conditions

**DOI:** 10.1111/pbi.12603

**Published:** 2016-08-17

**Authors:** Jinrui Shi, Huirong Gao, Hongyu Wang, H. Renee Lafitte, Rayeann L. Archibald, Meizhu Yang, Salim M. Hakimi, Hua Mo, Jeffrey E. Habben

**Affiliations:** ^1^ DuPont PioneerJohnstonIAUSA

**Keywords:** maize, ARGOS, CRISPR‐Cas9, genome editing, drought tolerance, grain yield

## Abstract

Maize *ARGOS8* is a negative regulator of ethylene responses. A previous study has shown that transgenic plants constitutively overexpressing *ARGOS8* have reduced ethylene sensitivity and improved grain yield under drought stress conditions. To explore the targeted use of *ARGOS8* native expression variation in drought‐tolerant breeding, a diverse set of over 400 maize inbreds was examined for *ARGOS8* mRNA expression, but the expression levels in all lines were less than that created in the original *ARGOS8* transgenic events. We then employed a CRISPR‐Cas‐enabled advanced breeding technology to generate novel variants of *ARGOS8*. The native maize GOS2 promoter, which confers a moderate level of constitutive expression, was inserted into the 5′‐untranslated region of the native *ARGOS8* gene or was used to replace the native promoter of *ARGOS*8. Precise genomic DNA modification at the *ARGOS8* locus was verified by PCR and sequencing. The *ARGOS8* variants had elevated levels of *ARGOS8* transcripts relative to the native allele and these transcripts were detectable in all the tissues tested, which was the expected results using the GOS2 promoter. A field study showed that compared to the WT, the *ARGOS8* variants increased grain yield by five bushels per acre under flowering stress conditions and had no yield loss under well‐watered conditions. These results demonstrate the utility of the CRISPR‐Cas9 system in generating novel allelic variation for breeding drought‐tolerant crops.

## Introduction

Developing more drought‐tolerant crops in a sustainable manner is one means to meet the demand of an increasing human population that will require more food, feed and fuel. Improvement in drought tolerance of crops is ultimately measured by an increase in grain yield under water‐limiting conditions. The physiological processes and metabolic networks underlying drought tolerance are complicated and often difficult to delineate. Nevertheless, the phytohormone ethylene is known to play an important role in regulating plant response to abiotic stress, including water deficits and high temperature (Hays *et al*., 2007; Kawakami *et al*., 2010, 2013). Field studies have shown that reducing ethylene biosynthesis by silencing *1‐aminocyclopropane‐1‐carboxylic acid synthase6* in transgenic maize plants improves grain yield under drought stress conditions (Habben *et al*., 2014). A higher yield also can be achieved by decreasing the sensitivity of maize to ethylene (Shi *et al*., 2015). *ARGOS* genes are negative regulators of the ethylene response and modulate ethylene signal transduction, enhancing drought tolerance when overexpressed in transgenic maize plants (Guo *et al*., 2014; Shi *et al*., 2015).

In addition to a transgenic approach, natural genetic variation for traits that impact drought tolerance has also been used in maize breeding programmes to improve grain yield. By applying precision phenotyping and molecular markers as well as understanding the genetic architecture of quantitative traits, maize breeders developed hybrids (AQUAmax^®^) with increased grain yield under drought stress conditions (Cooper *et al*., 2014; Gaffney *et al*., 2015). The drought tolerance in these hybrids is governed by multiple genes which individually have small effects. Potentially, some of these key genes could be identified and altered to generate new alleles to produce a larger effect, thus enhancing the breeding process. However, until recently, generating such allelic variation with physically or chemically induced mutagenesis was a random process, which made it difficult to produce intended DNA sequence changes at a target locus. In the past few years, efficient genome editing technologies have emerged, enabling rapid and precise manipulation of DNA sequences, and setting the stage for developing drought‐tolerant germplasm by editing major genes in their natural chromosomal context.

Four genome editing tools, meganucleases, zinc‐finger nucleases (ZFN), transcription activator‐like effector nucleases (TALEN) and the clustered regularly interspaced short palindromic repeat (CRISPR)/CRISPR‐associated nuclease protein (Cas) system, have provided targeted gene modification in plants (Čermák *et al*., 2015; Gao *et al*., 2010; Li *et al*., 2012, 2013; Shukla *et al*., 2009). Among these, the CRISPR‐Cas9 system is easiest to implement and is highly efficient. The system consists of a Cas9 endonuclease derived from *Streptococcus pyogenes* and a chimeric single guide RNA that directs Cas9 to a target DNA sequence in the genome. CRISPR‐Cas9 genome editing is accomplished by introducing a DNA double‐strand break in the target locus via Cas9, followed by DNA repair through either the endogenous imprecise nonhomologous end‐joining (NHEJ) or the high‐fidelity homology‐directed repair (HDR) pathways. NHEJ can induce small insertions or deletions at the repair junction while HDR stimulates precise sequence alterations, including programmed sequence correction as well as DNA fragment insertion and swap, when a DNA repair template is exogenously supplied. The system has been successfully tested in staple crops, such as maize, wheat, rice and soybean (Cai *et al*., 2015; Du *et al*., 2016; Jacobs *et al*., 2015; Jiang *et al*., 2013; Li *et al*., 2015; Liang *et al*., 2014; Shan *et al*., 2015; Sun *et al*., 2016; Svitashev *et al*., 2015; Wang *et al*., 2014; Zhang *et al*., 2014; Zhou *et al*., 2014, 2015).

In maize, endogenous *ARGOS8* mRNA expression is relatively low and spatially nonuniform. Previous field testing showed that constitutive overexpression of *ARGOS8* in transgenic plants increases grain yield under drought stress conditions without yield penalty in nonstress environments (Shi *et al*., 2015). Aiming at creating novel *ARGOS8* variants which would confer beneficial traits for maize breeding, the genomic sequence of *ARGOS8* was edited using CRISPR‐Cas‐enabled advanced breeding technology to produce ubiquitous and elevated expression across multiple tissues and at different developmental stages. Here, we report the generation of maize lines carrying *ARGOS8* genome‐edited variants and their hybrid yield performance in a field study. Our results demonstrate that modifying single native genes to change expression patterns can increase maize grain yield under drought stress conditions.

## Results

### Natural allelic variation of maize ARGOS8 and expression patterns

In wild‐type (WT) inbreds PH184C (proprietary) and B73 (public), *ARGOS8* mRNA expression is very low in all the tissues tested, ranging from 3 to 25 transcripts per ten million (TPTM), as measured with RNA sequencing (Figure S1). The only exception is in kernels where expression was approximately 260 TPTM. For comparison, the transcripts of the ubiquitously and moderately expressed *GOS2* gene, the maize homolog of rice *GOS2* (de Pater *et al*., 1992), are about 6000 TPTM in most tissues with the highest expression occurring in internodes (13 700 TPTM) and the lowest occurring in tassels (2500 TPTM). A survey of a diverse set of 419 proprietary and public inbred lines showed that the *ARGOS8* expression in leaves of 3‐week‐old seedlings only ranged from 0 to 20 TPTM (Figure S2), suggesting that the natural variation in expression levels among these inbreds was also low.

**Figure 1:**
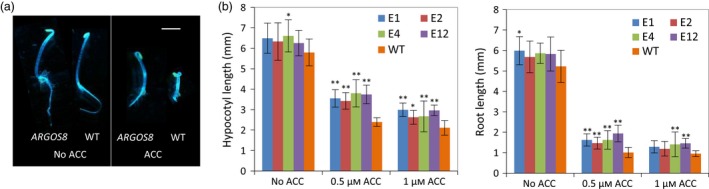
Maize *ARGOS8* reduces plant responses to ethylene when overexpressed in transgenic Arabidopsis plants. (a) Ethylene triple response of Arabidopsis *ARGOS8* transgenic plants (*ARGOS8*) and wild‐type (WT) controls to 0.5 μm of the ethylene precursor aminocyclopropane‐1‐carboxylic acid (ACC). A short version of *ARGOS8* was overexpressed under control of the cauliflower mosaic virus 35S promoter (35S). Composite image of representative 3‐day‐old etiolated seedlings. Bar = 2 mm. (b) Hypocotyl and root lengths of etiolated Arabidopsis seedlings overexpressing the short version of *ARGOS8*. Four transgenic lines (E1, E2, E4 and E12) and wild‐type (WT) controls were grown in the dark in the presence of indicated ACC concentrations for 3 days. Data are means ± SD,* n* = 15. Significant differences of the transgenic plants from the WT are denoted by asterisks (*P < 0.05, **P < 0.01, ANOVA, Tukey's HSD).

The protein encoded by the *ARGOS8* gene varies among inbred lines. The ARGOS8 protein in B73 has 118 amino acids (long version) while the protein from PH184C consists of 94 amino acids (short version). The difference in protein sequence is the presence of an N‐terminal extension of 24 amino acids in the long version. The extra coding sequence in the B73 allele is a result of a 7‐bp duplication in the 5′‐untranslated region (5′‐UTR) which produces an in‐frame ATG codon upstream of the original translation start codon. The 7‐bp duplication may be a footprint left behind by a transposon excision event (Scott *et al*., 1996). Like the long version from B73 (Shi *et al*., 2015, 2016), the short version of *ARGOS8* also reduces ethylene responses when overexpressed, as demonstrated in Arabidopsis transgenic plants. In the ethylene triple response assay (Bleecker *et al*., 1988), hypocotyls and roots of the etiolated 35S:*ARGOS8* Arabidopsis seedlings were longer than that in WT controls in the presence of the ethylene precursor aminocyclopropane‐1‐carboxylic acid (Figure 1). Although the short version of the ARGOS8 protein accumulated to a higher level than the B73 version in plants with a similar level of transcripts (data not shown), the native allelic variant still produces a very low level of the ARGOS8 protein in WT plants and it was not detectable by immunoblot analysis. This short allele, as well as the long B73 allele, was not able to confer drought‐tolerant phenotypes without ectopic overexpression. Consequently, this observed functional native diversity in the *ARGOS8* gene is not enough for targeted drought breeding.

### Novel ARGOS8 variants generated by the CRISPR‐Cas9 system

**Figure 2:**
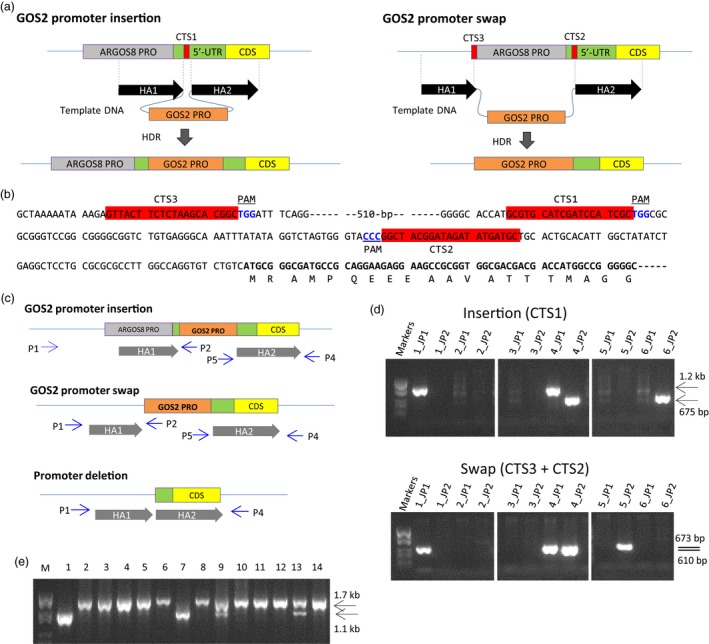
Editing the *ARGOS8* genomic sequence using the CRISPR/Cas9 system to generate variants with constitutive expression. (a) Schematic drawing illustrating the insertion of GOS2 PRO into the 5′‐UTR of *ARGOS8* and the promoter swap. CTS, CRISPR‐RNA target site; HA, homology arm; HDR, homology‐directed repair; GOS2 PRO, maize GOS2 promoter and the 5′‐UTR with an intron. (b) Genomic sequence of the *ARGOS8* 5′‐UTR and the upstream region. The CRISPR‐RNA target sites (CTS) are highlighted in red, and the protospacer adjacent motifs (PAM) are shown in blue font. The *ARGOS8* coding region is shown in bold font. (c) Diagram showing primers used in junction PCR for genotyping regenerated shoots and long PCR for amplifying and sequencing the entire modification region in homozygous plants. The relative position and direction of PCR primers (P) are indicated by arrows. P1 and P2 for the HR1 junction; P5 and P4 for the HR2 junction; P1 and P4 for the long PCR. (d) Junction PCR analysis of regenerated shoots. Agarose gel images are shown for representative regenerated shoots positive for one junction or two junctions and shoots negative in the junction PCR assay. JP1, HR1 junction PCR with the primer P1 and P2; JP2, HR2 junction PCR with P5 and P4. (e) PCR screening regenerated shoots for deletion in the *ARGOS8* locus. An agarose gel image is shown for PCR products amplified with the primer P1 and P4 in representative shoots (Lanes 1‐14) generated from the *CRISPR RNA‐3* and *RNA‐1* transformation. M, DNA molecular weight markers.

To achieve a moderate level of constitutive expression of *ARGOS8*, the maize GOS2 promoter and the 5′‐UTR with an intron, hereafter collectively referred to as GOS2 PRO, were either used to replace the native promoter of the *ARGOS8* gene or were inserted into the 5′‐UTR of *ARGOS8* in the inbred PH184C. Because both the promoter swap and insertion require precise manipulation of genomic DNA, we employed an RNA‐guided Cas9 endonuclease to generate DNA double‐strand breaks in a site‐specific manner, integrating the GOS2 PRO into the upstream region of *ARGOS8* via homology‐directed DNA repair (Figure 2a). A DNA repair template and genome editing reagents were delivered into immature embryos by particle bombardment and plantlets were regenerated from embryogenic calli. The reagents include an *S. pyogenes Cas9* gene and a single guide RNA (sgRNA) gene, *CRISPR RNA1* (*CR1*), in the GOS2 PRO insertion or two sgRNAs (*CR2* and *CR3*) in the GOS2 PRO swap (Figure S3), as well as *phosphomannose isomerase* (*PMI*), *ovule development protein2* (*ODP2*) and *WUSCHEL* (*WUS*) for stimulation of transformation and seedling regeneration (Svitashev *et al*., 2015). The DNA repair template consisted of the GOS2 PRO flanked by two DNA fragments of approximately 400‐bp homologous to genomic sequences immediately adjacent to the Cas9 cleavage sites in the *ARGOS8* locus (Figure 2b). Of approximately 1000 immature embryos particle‐bombarded for the promoter insertion and swap experiments, 194 and 334 shoots, respectively, were regenerated on the selection medium (Table S1). To eliminate the shoots whose CRISPR RNA target sites (CTS) were not altered, a rapid screening was performed using a quantitative PCR (qPCR) assay (Table S2), which estimates the copy number of CTS. Shoots with no modification at CTS contained two copies of the wild‐type CTS, shoots with CTS modification in one of the two sister chromosomes had one intact copy, while modification in both chromosomes would reduce the copy number to zero. With this screening, 190 and 172 regenerated shoots from the insertion and swap experiments, respectively, were selected for genotyping with a junction PCR assay.

A pair of junction PCR assays was designed to detect GOS2 PRO inserts or swaps at CTS due to homologous recombination (Figure 2c). In the insertion experiments, five of the 190 shoots from the initial screening were found positive for one of the two junctions, and two shoots were positive for both junctions (Figure 2d and Table S1). These shoots were transferred to rooting media, and three plantlets were regenerated. Genotyping the T0 plants with the junction PCR assays revealed that one plant contained the GOS2 PRO insert in the *ARGOS8* locus. The junction PCR products were sequenced, and expected sequences were confirmed (Figure S4). This line is referred to as *ARGOS8‐variant1* (*ARGOS8‐v1*). For the GOS2 promoter swap, 23 of the 172 shoots obtained from the initial screening were positive for at least one junction. Among them, three were positive for both junctions (Figure 2d and Table S1). From these shoots, eight plantlets were regenerated. Of these T0 plants, two produced expected junction PCR products for the promoter swap in the *ARGOS8* locus. Sequencing the PCR products confirmed correct sequences from both junctions (Figure S4). One of the lines is referred to as *ARGOS8‐variant2* (*ARGOS8‐v2*). Genotyping also revealed that the *ARGOS8‐v1 and ARGOS‐v2* were heterozygous.

**Figure 3:**
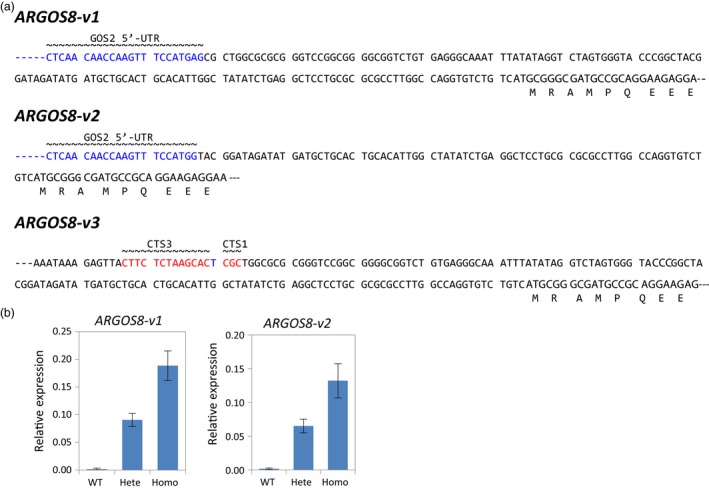
Maize genome‐edited *ARGOS8* variants. (a) Genomic sequence upstream of the *ARGOS8* coding region in three genome‐edited variants. The entire modification region in homozygous F2 plants was amplified using long PCR, and the PCR products were sequenced. Part of the GOS2 5′‐UTR sequence (blue font) and the remaining 5′‐UTR of *ARGOS8* as well as the 5′‐terminus of *ARGOS8* coding sequence are shown. In the promoter deletion variant *ARGOS8‐v3*, the remnant CTS3 and CTS1 sequences are highlighted. (b) Relative expression levels of *ARGOS8* in leaves as measured by qRT‐PCR. Means ± SD are shown for F2 plants of 14‐day‐old *ARGOS8‐v1* and 18‐day‐old *ARGOS8‐v2*;* n* = 10–24. WT, wild‐type; Hete, Heterozygote; Homo, homozygote.

F1 seeds of *ARGOS8‐v1* and *ARGOS8‐v2* were produced by crossing the T0 plants with WT PH184C plants. F1 plants were genotyped by PCR to select those that carry the *ARGOS8‐v1* or *ARGOS8‐v2*, but were nulls for the genome editing reagents *Cas9*,* sgRNA*,* PMI*,* ODP2* and *WUS*. To eliminate the plants containing random insertions of the DNA repair template, qPCR was performed to assess the copy number of the GOS2 PRO and *ARGOS8*. Selected clean F1 plants were backcrossed to produce BC1 seeds, or self‐pollinated to obtain F2 seeds. Among the F2 segregants, homozygous plants were used to determine the sequence integrity of the newly created *ARGOS8* variants. The entire genomic region was amplified using long PCR with primers P1 and P4, which were derived from genomic sequences further upstream and downstream of the homology arms used in the DNA repair templates (Figure 2c). Sequencing the long‐PCR products confirmed that the *ARGOS8‐v1* and *ARGOS8‐v2* possess the expected DNA sequences (Figure 3a). The *ARGOS8‐v1* and *ARGOS8‐v2* segregated in a Mendelian fashion in BC1 and F2 populations (data not shown). Quantitative reverse‐transcription PCR (qRT‐PCR) analysis showed that the abundance of *ARGOS8* transcripts in leaves of homozygous plants is approximately twice as much as in the heterozygotes for both lines (Figure 3b).

**Figure 4:**
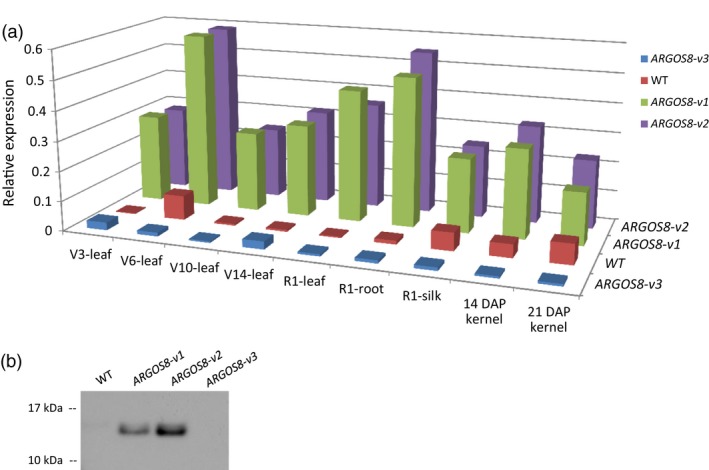
Comparison of the *ARGOS8* expression in genome‐edited variants and wild‐type maize plants. (a) Relative expression of *ARGOS8* in a selection of maize tissues and stages. mRNA was quantified with qRT‐PCR. Six individual plants were analysed for the genome‐edited variants and two plants for WT controls. DAP, days after pollination. (b) ARGOS8 protein expression in developing kernels. Immature kernels (21 DAP) were analysed by immunoblotting using a monoclonal anti‐ARGOS8 antibody.

To obtain controls for analysing *ARGOS8* gene expression in the *ARGOS8* variants, the shoots regenerated from particle‐bombarded immature embryos were screened for ARGOS8 promoter deletions in the promoter swap experiments using *CR3* and *CR1*. Of 185 shoots screened, 30 produced a PCR product shorter than that expected for WT plants (Figure 2c and e), indicating a deletion between CTS3 and CTS1. Sequencing the PCR products from two T0 plants showed that both had an extra base pair (one line having T and the other A) at the junction of the nonhomologous end‐joining (Figures 3a and S4). Similarly, approximately 13% (23 of 176) of the regenerated shoots were found contain deletion at the target sites in the swap experiments using *CR3* and *CR2* (data not shown). The deletion of the 550‐bp genomic DNA fragment between CTS3 and CTS1 removed part of the *ARGOS8* 5′‐UTR and the upstream promoter sequence (Figures 2b and 3a). One of the lines was referred to as *ARGOS8‐variant3* (*ARGOS8‐v3*). The *ARGOS8* transcripts and proteins were undetectable in *ARGOS8‐v3* (Figure 4a and b). The line had normal growth and development, and no phenotypic defects were observed under normal growing conditions, indicating that the *ARGOS8* gene is likely dispensable.

### Expression patterns of ARGOS8 in genome‐edited variants

The mRNA expression of *ARGOS8* in the genome‐edited variants was analysed in leaves, roots, silks and kernels using qRT‐PCR. In the uppermost collared leaves of plants at the developmental stages V3, V6, V10 and V14, the *ARGOS8* transcript level in *ARGOS8‐v1* and *ARGOS8‐v2* was significantly higher than that in WT plants with the highest expression found at V6 (Figure 4a). At the developmental stage R1, silks, roots and leaves all had higher levels of the *ARGOS8* mRNA in the genome‐edited plants relative to WT controls. In developing kernels 14 and 21 days after pollination (DAP), the *ARGOS8* mRNA was also more abundant in the *ARGOS8‐v1* and *ARGOS8‐v2* than the WT. The ARGOS8 protein was detectable by immunoblot analysis in the developing kernels of the genome‐edited variants, but not in the WT (Figure 4b). The two variants had a similar level of *ARGOS8* mRNA expression in all the tissues tested (Figure 4b).

### Improved grain yield under drought stress environments

The two genome‐edited variants *ARGOS8‐v1* and *ARGOS8‐v2* were crossed with an inbred tester to create a hybrid for field evaluation. These variants were compared to a wild‐type hybrid that had not undergone genome editing. Entries were evaluated across multiple environments at eight locations throughout the United States. At the end of the growing season, locations were grouped into three environmental types based on the occurrence of drought stress. Four locations had yields near or above 200 bushel per acre; these were classified as optimal locations (OPT) where water deficits were not a constraint. The remaining locations were grouped as either flowering stress (FS) or grain‐filling stress (GFS), based on the EnClass location classification system (Loffler *et al*., 2005).

Significant differences among entries were observed for grain yield in the FS location group, with the *ARGOS8‐v1 and ARGOS8‐v2* entries yielding approximately five bushel per acre more than the control (Table 1). In contrast, there was no significant difference in grain yield between the variants and WT in the GFS or OPT locations (Table 1). The GFS locations were characterized by limited soil moisture availability due to soil texture, and drought stress developed very quickly. This may have resulted in early cessation of grain filling in the *ARGOS8‐v1 and ARGOS8‐v2* entries; grain moisture was significantly less than in the control for *ARGOS8‐v1* (Table S3). Plant height and ear height increased by a small (2.6 and 3.2 cm, respectively) but significant amount in the *ARGOS8‐v2* (Table S3) in the OPT locations. No differences were observed in thermal time to silk or to shed.

**Table 1:** Grain yield of *ARGOS8* genome‐edited variants and wild type under flowering stress, grain‐filling stress and optimal (well‐watered) conditions

	Flowering	Grain‐filling	Optimal
	Stress	Stress	
	ton ha^−1^ (bushel acre^−1^)		
*ARGOS8‐v1*	8.67 (138.0)^a^	7.47 (119.0)	13.13 (209.0)
*ARGOS8‐v2*	8.67 (138.0)^a^	7.54 (120.0)	13.19 (210.0)
WT	8.34 (132.8)	7.72 (122.9)	13.01 (207.1)

Data are from two individual genome‐edited variants (*ARGOS8‐v1*,* ARGOS8‐v2*) and wild type tested as one hybrid at eight locations in 2015. Predicted difference for each variant is compared with the wild type. All analyses were implemented using ASReml with output of the model presented as best linear unbiased predictions (see Experimental procedures).

a Predicted difference significant at *P* < 0.1.

## Discussion

Constitutive overexpression of *ARGOS8* using a transgenic approach increases grain yield in maize under drought stress conditions (Shi *et al*., 2015). To explore the feasibility of recapitulating the *ARGOS8* transgene effect using a conventional breeding approach, we determined the native expression levels of *ARGOS8* in a set of public and proprietary maize inbred lines. None of the inbreds we examined had mRNA expression levels great enough to match those in the transgenic events. In addition, a naturally occurring variant of *ARGOS8* encoding a shorter protein also was not able to confer desired phenotypes without overexpression. Therefore, conventional breeding with this gene was not deemed worthwhile, and we elected to use a CRISPR‐Cas enabled advanced breeding technology to generate new *ARGOS8* variants by changing the DNA sequence at the native *ARGOS8* locus. Replacement of the ARGOS8 promoter with a maize GOS2 promoter (GOS2 PRO), or insertion of a GOS2 PRO into the 5′‐UTR of the *ARGOS8* gene, led to a change in the *ARGOS8* expression pattern from tissue preferred to ubiquitous, and from relatively low mRNA expression levels to significantly increased *ARGOS8* expression levels. Precise modification of the nucleotide sequence of *ARGOS8* at its native location in the genome was achieved, as determined by PCR assays of the entire region followed by sequencing. The *ARGOS8* variants were found to be stably inherited via analysis of over four generations. Field testing showed that the novel *ARGOS8* variants increased grain yield under drought stress conditions. These yield results are similar to previous results obtained from transgenic plants overexpressing *ARGOS8* (Shi *et al*., 2015). These results demonstrate the utility of genome editing in creating novel allelic variation for enhancing crop drought tolerance.

The mutation rate at CRISPR‐RNA target sites in the regenerated shoots ranged from 60% to 98%, similar to that reported in maize gene modification studies using stably transformed lines (Svitashev *et al*., 2015). In the promoter swap experiments using two guide RNAs, we observed a frequency of approximately 16% (30 of 185) for DNA fragment deletion due to the nonhomologous end‐joining. The homology‐directed DNA swap at the *ARGOS8* locus occurred in approximately 1% (3 of 334) of the regenerated shoots. A comparable frequency (2 of 194) was found for insertion when one guide RNA was used. In a previous study, the insertion frequency at the maize *liguleless1* locus was 2.5%–4.1% (Svitashev *et al*., 2015). CRISPR‐RNA target sites, the surrounding genomic DNA sequences, insert sequences and the genotype of host plants as well as other factors may contribute to the difference in insertion frequencies. We also observed that nearly 60% (19 of 30; Table S1) of the regenerated shoots failed to produce T0 plants. Of the five two‐junction‐positive events identified in the shoot stages, only three T0 plants were recovered, indicating more genome‐edited lines can be obtained by improving maize inbred transformation.

The maize *ARGOS8* gene is a negative regulator of ethylene responses. ARGOS8 proteins physically interact with the ethylene receptor signalling complex, modulating ethylene perception and the early stages of the ethylene signal transduction (Shi *et al*., 2016). Transgenic maize and Arabidopsis plants overexpressing *ARGOS* genes exhibit reduced sensitivity to ethylene (Rai *et al*., 2015; Shi *et al*., 2015), enhanced cell elongation and/or division resulting in taller plants, larger leaves, and longer ears in maize (Guo *et al*., 2014; Shi *et al*., 2015), as well as creating larger organs in other plant species (Feng *et al*., 2011; Hu *et al*., 2003; Kuluev *et al*., 2011). Drought stress often reduces plant growth and can adversely affect development, leading to grain yield loss in crops. These stress‐induced changes at the whole plant level are largely due to reduced cell number and/or size. Constitutively overexpressed ARGOS likely counteracts the effect of water deficiency by promoting cell expansion and/or division, mitigating the yield loss by enhancing plant growth under drought stress. Here, we show that maize variants of the *ARGOS8* gene generated by altering its regulatory elements can deliver a significant increase in grain yield under a flowering stress condition with no yield loss under an optimal condition, similar to that of *ARGOS8* transgenic plants (Shi *et al*., 2015). However, this was not the case when variants were exposed to a grain‐filling stress (Table 1). This result was not surprising given that much of the yield increase resulting from ectopic expression of *ARGOS8* under abiotic stress comes from an increase in kernel set (Shi *et al*., 2015), which is primarily determined at flowering time.

Unlike transgenic *ARGOS8* plants, the maize inbred lines carrying the genome‐edited variants contain no transformation selection markers or any nonmaize DNA. All the reagents (*i.e*. helper genes) used in maize DNA sequence editing, including *Cas9*,* sgRNA*,* PMI*,* ODP2* and *WUS* as well as plasmid backbones, were not required for the function of newly generated *ARGOS8* variants and were removed by backcrossing in the early stages of breeding. Instead of *Agrobacterium*‐mediated transformation, particle bombardment was employed to deliver the genome editing reagents; thus, no plant pathogen was involved in the generation of these variants. The DNA repair template (GOS2 promoter flanked by homology arms) originated from maize genomic DNA; only the maize GOS2 promoter is site‐specifically integrated into the *ARGOS8* locus via homologous recombination, leading to the designed modification of *ARGOS8* expression.

The *ARGOS8* editing process can be summarized as a two‐step procedure: duplication of the GOS2 promoter and translocation to the *ARGOS8* locus. Both duplication and translocation of DNA fragments occur naturally in maize (Wang *et al*., 2015; Zhang *et al*., 2000). During its evolutionary history, the maize genome has undergone several rounds of whole‐genome duplication. In addition, segmental duplication, which involves DNA fragments of different sizes ranging from a few base pairs up to many megabases which may or may not contain intact, functional genes, also play an important role in shaping the maize genome and increasing genetic diversity (Lai *et al*., 2004; Schnable *et al*., 2009). Similarly, this process occurs naturally in other plants and animals, including humans (Zhang *et al*., 2005). Comparative genomic studies have shown that although segmental duplication drives the formation of clusters of closely related genes, duplicated sequences can translocate to different chromosomal locations, resulting in dispersal of paralogs throughout the genome (Freeling *et al*., 2008; Lai *et al*., 2004; Mendivil Ramos and Ferrier, 2012). For example, the plant disease resistance NBS‐LRR genes are particularly prone to being transposed (Ameline‐Torregrosa *et al*., 2008; Baumgarten *et al*., 2003; Leister *et al*., 1998; Richly *et al*., 2002), vividly attesting to segmental duplication and translocation occurring in plants. This natural rearrangement of DNA fragments occurs spontaneously at a low frequency in maize and has been exploited by breeders over the decades for maize improvement. Similarly, the CRISPR‐Cas‐enabled advanced breeding technology allows precise integration of duplicated genetic elements into a target locus and can enhance the grain yield of maize.

Since the adoption and widespread use of hybrid maize, natural allelic variations in a large number of genes, each with small effects, have improved drought tolerance, even though it has been suggested that the stress‐tolerant alleles are present at relatively low frequencies in most elite breeding populations (Blum, 1988). With increasing knowledge on plant response to drought stress and molecular understanding of gene networks underlying the physiological processes that impact drought tolerance, monogenic drought tolerance in maize has become possible by a transgenic approach. Indeed, there are multiple examples of the validation of the efficacy of transgenes in elite hybrids under field conditions (Castiglioni *et al*., 2008; Guo *et al*., 2014; Habben *et al*., 2014; Leibman *et al*., 2014; Nuccio *et al*., 2015; Shi *et al*., 2015). With the advent of CRISPR‐Cas enabled advanced technology, a new technique is now available to provide new sources of genetic variation for plant breeding. This genome editing study of *ARGOS8* demonstrates that single endogenous genes can be modified to create novel variants that have a significantly positive effect on a complex trait such as drought tolerance. In short, the generation and use of genome‐edited variants is a seminal addition to the precision breeding toolbox that can enhance changes to the plant genome in a predictable manner.

## Experimental procedures

### Plasmids and reagents used for plant transformation

Plasmids were designed to insert a DNA fragment into the 5′‐UTR of target genes using a single guide RNA (sgRNA), the Cas9 endonuclease gene derived from *Streptococcus pyogenes* and a DNA repair template. For the DNA fragment swap, two sgRNA were used. The sgRNA gene was adapted from Mali *et al*. (2013) and consists of a maize U6 polymerase III promoter (Svitashev *et al*., 2015), a CRISPR RNA (crRNA), a transactivating CRISPR RNA (tracrRNA) and a terminator (Figure S3). The Cas9 expression cassette contains the maize *UBIQUITIN1* promoter and potato protease inhibitor II terminator. The Cas9 sequence was maize codon optimized and added the potato ST‐LS1 intron as well as the nuclear localization signals from the SV40 and the *Agrobacterium tumefaciens* Vir D2, as previously described (Svitashev *et al*., 2015), for appropriate expression and nuclear targeting in maize. The DNA repair template plasmid carries the maize GOS2 promoter (NCBI GenBank accession no. GQ184457; nucleotides 218 974–220 796 in reverse direction; Barbour *et al*., 2003) that was inserted between two homology arms each approximately 400 bp derived from the genomic sequence flanking the CRISPR‐RNA target site (CTS; Figure 2a). The vector also contains a multiple cloning site of 61‐bp immediately upstream of the GOS2 promoter. Constructs were assembled using chemically synthesized DNA fragments with standard DNA techniques (Sambrook *et al*., 1989). To facilitate delivery of the genome editing reagents into maize cells and regeneration of plants, three expression cassettes encoding transformation selection marker phosphomannose isomerase (PMI) as well as cell division and callus growth‐promoting proteins ovule development protein2 (ODP2) and WUSCHEL (WUS) were constructed as previously described (Ananiev *et al*., 2009; Svitashev *et al*., 2015).

### Maize transformation

Biolistic‐mediated transformation of maize immature embryos was performed according to Svitashev *et al*. (2015). Briefly, gold particles, 0.6 μm in diameter, were washed with cold, 100% [v/v] ethanol and sterile distilled water. The DNA purified with QIAprep Spin Miniprep (Qiagen) was precipitated on the washed gold particles using a water‐soluble cationic lipid TransIT‐2020 (Mirus). Fifty microlitres of gold particles (water solution of 10 mg/mL) and 1 μL of TransIT‐2020 water solution were added to the premixed DNA, mixed gently and incubated on ice for 10 min. DNA‐coated gold particles were then centrifuged at 8 000 g for 1 min. The pellet was rinsed with 100 μL of 100% [v/v] ethanol and resuspended by a brief sonication. Immediately after sonication, DNA‐coated gold particles were loaded onto the centre of a macrocarrier (10 μL of each) and allowed to air dry. Immature embryos 9–11 days after pollination were bombarded using a PDS‐1000 Helium Gun (Bio‐Rad) with a rupture pressure of 425 psi. Postbombardment culture, selection and plant regeneration were carried out as previously described (Gordon‐Kamm *et al*., 2002). Regenerated shoots were sampled for initial qPCR screening and junction PCR. Leaf discs were taken from T0 seedlings 2 weeks post‐transplanting for genotyping. F1 seeds were produced by crossing the T0 with wild‐type plants. BC1 and F2 seeds were produced by backcrossing and self‐pollination, respectively.

### Arabidopsis transformation and ethylene response assay

The *35S::ARGOS8* construct was assembled and transgenic Arabidopsis plants generated as described (Shi *et al*., 2015). Transgenic lines were selected based on the expression of the fluorescent marker yellow fluorescence protein in T1 seeds. The *ARGOS8* transgene expression was confirmed by qRT‐PCR. To determine the activity of *ARGOS8* in reducing plant response to ethylene, the ethylene triple response assay (Bleecker *et al*., 1988) was carried out in the presence of the ethylene precursor aminocyclopropane‐1‐carboxylic acid (ACC). Surface‐sterilized and stratified seeds were germinated in the dark for 3 days in agar that contained one‐half‐strength Murashige and Skoog salts and 1% [w/v] sucrose supplemented with 0, 0.5, or 1 μm ACC. Hypocotyl and root lengths of the etiolated seedlings were measured by photographing the seedlings with a digital camera and using image analysis software ImageJ (National Institutes of Health).

### DNA extraction, PCR genotyping and sequencing

DNA was extracted from regenerated shoots or leaf discs as described in Gao *et al*. (2010). PCR was performed using REDEtract‐N‐Amp PCR readyMix (Sigma) or Phusion High‐Fidelity PCR Master Mix (NEB) according to the manufacturer's instructions. Quantitative PCR (qPCR) was carried out as described in Svitashev *et al*. (2015). The primer sequences used in PCR and qPCR are listed in Supporting Information Table S2. The PCR products were sequenced directly or cloned into the pCR2.1‐TOPO vectors (Thermo Fisher Scientific) before sequencing.

### RNA extraction, qRT‐PCR and RNA sequencing

Total RNA was isolated with Qiagen RNA Isolation Kit (Qiagen). The DNaseI Enzyme Kit (Roche) was used to remove DNA from the RNA samples. Complementary DNA was synthesized from the total RNA using the High Capacity cDNA Reverse Transcription Kit (Thermo Fisher Scientific). PCR amplifications were performed using the TaqMan probe‐based detection system according to the manufacturer's instructions (Applied Biosystems). Primers and probes are shown in Table S2. Relative quantification values were determined using the difference in Ct from the target genes and the reference gene, maize *UBIQUITIN5*.

RNA sequencing (RNA‐seq) was performed as described (Shi *et al*., 2015). In brief, total RNA was isolated from frozen maize tissues and used to prepare sequencing libraries using the TruSeq mRNA‐Seq Kit (Illumina), and sequenced on the Illumina HiSeq 2000 system with Illumina TruSeq SBS v3 reagents. On average, 10 million sequences were generated for each sample. The resulting sequences were trimmed based on quality scores and mapped to the maize B73 reference genome sequence V2 and normalized to reads per kilobase of transcript per ten million mapped reads. The generated data matrix was visualized and analysed in GeneData Analyst software (Genedata AG, Basel, Switzerland).

### Immunoblot analysis

To detect ARGOS8 proteins, extracts were prepared from immature kernels, proteins separated by SDS‐PAGE, blotted to a nitrocellulose membrane and probed with a monoclonal anti‐ARGOS8 antibody. The primary antibodies were detected with a HRP‐conjugated goat anti‐mouse secondary antibody and the Pierce SuperSignal^®^ West Dura Extended Duration Substrate (Thermo Fisher Scientific).

### Maize hybrid yield testing

To evaluate the genome‐edited variants, field trials were conducted across multiple environments in small plots (approximately 4 m^2^) with 2–4 replications at each of eight locations. Hybrid seed for these trials was generated by crossing the genome‐edited *ARGOS8* variants with an inbred tester. A wild‐type hybrid containing the native *ARGOS8* allele served as the comparator. The experimental variants and control were grown in field environments at research centres in Woodland, CA; Garden City, KS; Plainview, TX; York, NE; Marion, IA; Johnston, IA; and Princeton, IN in 2015. Some environments were managed to impose various levels of drought stress while others were managed for optimum yield/nonstress conditions. Fertilizer at each location was applied to achieve maximum yields. Weeds and pests were controlled according to local practices. Grain mass and grain moisture data were collected using a small plot combine. Grain yield was adjusted to a constant 15% moisture. Additional agronomic characteristics evaluated at selected locations were plant and ear height and thermal time to shed and silk.

The field experimental design was set up as a randomized complete block arrangement. Data analysis was by ASREML (VSN International Ltd), and the values reported are BLUPs (Best Linear Unbiased Predictions; Cullis *et al*., 1998; Gilmour *et al*., 2009). A mixed‐model framework was used to perform the analysis. The model included replicate, row, column and heterogeneous residual variance with a separable autoregressive correlation for both row and column directions (AR1*AR1) within each location to reduce the impact of spatial variation in the field. In the analysis, the main effect of location type was considered a fixed effect. The main effect of entry and its interaction with location type were considered random effects. Statistical significance is reported with a *P*‐value of 0.1 in a two‐tailed test.

## Conflict of Interest

The authors are employees of DuPont Pioneer.

## Supporting information


**Figure S1** Maize *ARGOS8* gene expression. The transcript abundance of *ARGOS8* in various tissues of maize inbred PH184C was measured by RNA sequencing. Samples were taken from the plants at the developmental stage of V10, VT/R1 and R4. TPTM, transcript per ten million.
**Figure S2** Comparison of *ARGOS8* gene expression among 419 maize inbred lines. The *ARGOS8* transcript levels in leaves of 3‐week‐old seedlings were measured by RNA sequencing. TPTM, transcript per ten million.
**Figure S3** Single guide RNA genes used in editing the genomic sequence of *ARGOS8*. (a) Scheme illustrating the structure of a single guide RNA (sgRNA) gene. (b) DNA sequences of the transcription region in the sgRNA genes *CRISPR RNA1 (CR1)*,* CR2 and CR3*. U6 PRO, maize U6 polymerase III promoter; crRNA, CRISPR RNA (in red font) functioning as a guide; tracrRNA, transactivating CRISPR RNA, the sgRNA scaffold.
**Figure S4** DNA sequences of the junction PCR products amplified from *ARGOS8* variants.
**Table S1** Frequency of the GOS2 promoter insertion with one target site and the promoter swap with two target sites in T0 maize plants.
**Table S2** Primers and probes used for generation and characterization of *ARGOS8* genome editing variants.
**Table S3** Plant height (PLTHT), ear height (EARHT) and grain moisture (MST) of *ARGOS8* genome‐edited variants and wild type under flowering stress, grain‐filling stress and optimal (well‐watered) conditions.
