# Supplementary material for: ARGOS8 variants generated by CRISPR‐Cas9 improve maize grain yield under field drought stress conditions
Source: Plant Biotechnol J. 2016 Aug 17;15(2):207–16. doi: 10.1111/pbi.12603 (PMC5258859; doi:10.1111/pbi.12603)
Supplement: Supplementary file 1 — Figure S1 Maize ARGOS8 gene expression. The transcript abundance of ARGOS8 in various tissues of maize inbred PH184C was measured by RNA sequencing. Samples were taken from the plants at the developmental stage of V10, VT/R1 and R4. TPTM, transcript per ten million. Figure S2 Comparison of ARGOS8 gene expression among 419 maize inbred lines. The ARGOS8 transcript levels in leaves of 3‐week‐old seedlings were measured by RNA sequencing. TPTM, transcript per ten million. Figure S3 Single guide RNA genes used in editing the genomic sequence of ARGOS8. (a) Scheme illustrating the structure of a single guide RNA (sgRNA) gene. (b) DNA sequences of the transcription region in the sgRNA genes CRISPR RNA1 (CR1), CR2 and CR3. U6 PRO, maize U6 polymerase III promoter; crRNA, CRISPR RNA (in red font) functioning as a guide; tracrRNA, transactivating CRISPR RNA, the sgRNA scaffold. Figure S4 DNA sequences of the junction PCR products amplified from ARGOS8 variants. Table S1 Frequency of the GOS2 promoter insertion with one target site and the promoter swap with two target sites in T0 maize plants. Table S2 Primers and probes used for generation and characterization of ARGOS8 genome editing variants. Table S3 Plant height (PLTHT), ear height (EARHT) and grain moisture (MST) of ARGOS8 genome‐edited variants and wild type under flowering stress, grain‐filling stress and optimal (well‐watered) conditions. [file PBI-15-207-s001.pdf]

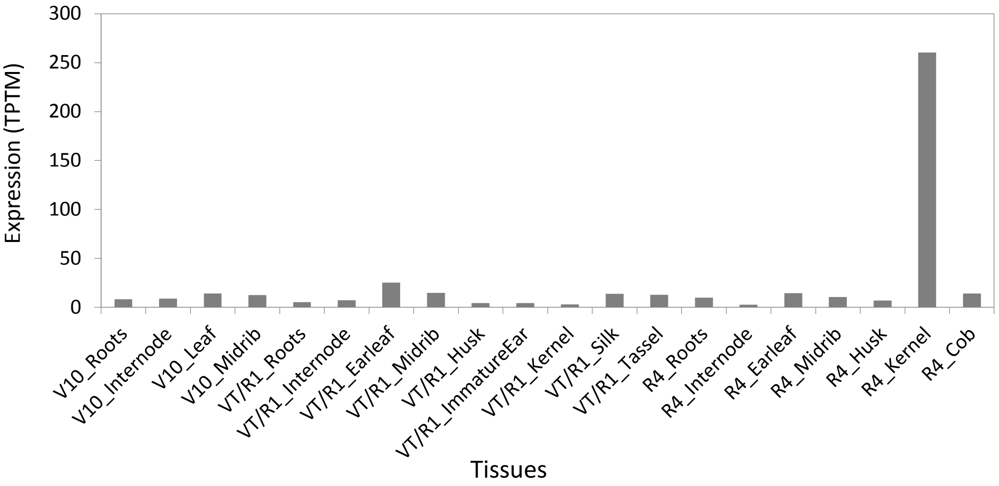

Figure S1. Maize *ARGOS8* gene expression. The transcript abundance of *ARGOS8* in various tissues of maize inbred PH184C was measured by RNA sequencing. Samples were taken from the plants at the developmental stage of V10, VT/R1 and R4. TPTM, transcript per ten million.

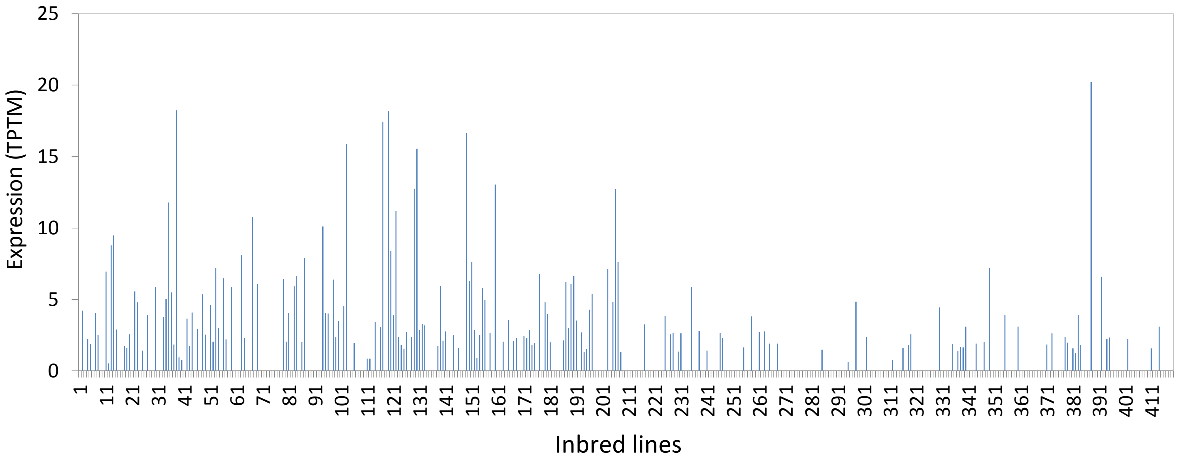

Figure S2. Comparison of *ARGOS8* gene expression among 419 maize inbred lines. The *ARGOS8* transcript levels in leaves of 3-week-old seedlings were measured by RNA sequencing. TPTM, transcript per ten million.

(a)

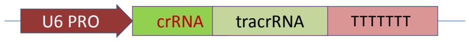

(b)

CR1

GCGTGCATCG ATCCATCGCG TTTTAGAGCT AGAAATAGCA AGTTAAAATA AGGCTAGTCC GTTATCAACT TGAAAAAGTG GCACCGAGTC GGTGCT

CR2

GCATCATATC TATCCGTAGC CGTTTTAGAG CTAGAAATAG CAAGTTAAAA TAAGGCTAGT CCGTTATCAA CTTGAAAAAG TGGCACCGAG TCGGTGCT

CR3

GTTACTTCTC TAAGCACGGC GTTTTAGAGC TAGAAATAGC AAGTTAAAAT AAGGCTAGTC CGTTATCAAC TTGAAAAAGT GGCACCGAGT CGGTGCT

Figure S3. Single guide RNA genes used in editing the genomic sequence of *ARGOS8.* (a) Scheme illustrating the structure of a single guide RNA (sgRNA) gene. (b) DNA sequences of the transcription region in the sgRNA genes *CRISPR RNA1 (CR1)*, *CR2 and CR3*. U6 PRO, maize U6 polymerase III promoter; crRNA, CRISPR RNA (in red font) functioning as a guide; tracrRNA, trans-activating CRISPR RNA, the sgRNA scaffold.

>ID189_HR1 junction PCR product (P1/P2), *ARGOS8-v1*
GCGCCATTCCCTAAAGGTAACTACTAAAACAGAACACAATTTTCTTAGGCCATCTCCAGCAGGCTGTGTAAACAGCCGTGCAAAATGTTGTTTTGCA
CTGTTTGTACAGTGAAGTTTTAAATAAAGAGTAAGATAGGAAATGAGATAAAGAGTATTCTGGAGATAGTCTTATACATCACCGTAGCAACGCATT
AATATATACCTAGTTAATATTATATGATTTAAATTTTATCTTAAAATACTTGACGCTCTATGAGTTTAGTAGTTTATAATACATATTTATCTTCTTCTAA
TACATGAACATCCTTTTTAGAATACGTCATAGGACGATCACCAGTACGGAGGATCTAGGCAAACAACATAAGAATCGAGCGAGTGCTAGGAGCATC
TCTAAAAGACTAATAAAATAACTCGTAAAGTTAAATTTTAGATACTCAAGAGTAAAATAACTATTTAACAGACTCGTTATTTGACTCATCAATATACC
CTTCAAATTGACTCTCTCACTATCTAAACTTGGCTAGCCACTATGACTAACTAAACTAGATAGATATTTTGGTGGAGTAAGATGCTATATATAAAGTG
TAATATTTATAAAATGATAAATAAAAAGCTAAAAATAAAGAGTTACTTCTCTAAGCACGGCTGGATTTCAGGCCTCTAGTCCTCTACTAGTACTAGCT
ACACGACGTGCACGCATGCATCACAGCATCAACAACTAGACACGCACACGCTGCACGCGGCCGGGGAACCCACTGATTCCCCCCTTCCCCGCGCGC
GGTTTGATTTCCTTTCCTGGTACGGATCCATATCTGAGGGCTTGTTCGGTTATTCCCAACACACATGTATTGGATGGGATTGAAAAAAAAATGAGAA
GAAGTTTGACTTGTTTGGGATTCAAACCCATCCAATCCCACTCAATCCACATGGATTGAGAGCTAACCGAACAAGCCCTCATAGTACATACCTGGTA
CGGATCCATATCATAGTACATAGATCCAGTAGAATAGAAGGTGATCCGACCGCCGGCGCTTGCGTTGTTTTCCCCGGTCCATTGAACCTGCCAACCC
TCCTAACCACAGGCACGCCAAACCGCGGGCTCCGGCCACCACCGCCACCGCCACCTGCCCTGCCGCACCTCTCCAACCCCAAATCCAGGGGGGGGG
GGGGGCACCATGCGTGCATCGATCCATGCTCGTGTCCAAGCGTCACTTACGATTAGC

>ID189_HR2 junction PCR product (P5/P4), *ARGOS8-v1*
ACCGTCCTTATCTCTGCATCATCTATCTGTGCTAGTTTCTGCGTGCTTCTCAAATATTTCCGGCCTGTGTAGCATGTGACTGATAATATGATTTTGGCA
GCTTCTGCATAAGAACAACAAATCAAAAGCTTGATCAGCTCGGTGCCTACAAAACCTCAACAACCAAGTTTCCATGAGCGCTGGCGCGCGGGTCCG
GCGGGGCGGTCTGTGAGGGCAAATTTATATAGGTCTAGTGGGTACCCGGCTACGGATAGATATGATGCTGCACTGCACATTGGCTATATCTGAGG
CTCCTGCGCGCGCCTTGGCCAGGTGTCTGTCATGCGGGCGATGCCGCAGGAAGAGGAAGCCGCGGTGGCGACGACGACCATGGCCGGGGGCAA
GGTGGCGGCGCTGCTGGCCACGGCGGCCGCGCTGCTGCTGCTGCTCCCGCTGGCGCTGCCGCCGCTGCCGCCGCCGCCCACGCAGCTGTTGTTCG
TCCCCGTGGTCTTGCTGCTCCTCGTGGCGTCCCTCGCGTTCTGCCCCGCCGCGACCCCCTCGCCGTCGCCGATGCATGCCGCCGACCACGGGTCGTT
CGGGACCACTGGATCACCGCACCTATGTTGACGCTGACGATGAGAGATCTCGTCGATCGACGGTCGGCTTGCAATGGCGCGTCGGAATCAAGCAG
TTCGCACACAC

>ID664_HR1 junction PCR product (P1/P2), *ARGOS8-v2*
GCGCCATTCCCTAAAGGTAACTACTAAAACAGAACACAATTTTCTTAGGCCATCTCCAGCAGGCTGTGTAAACAGCCGTGCAAAATGTTGTTTTGCA
CTGTTTGTACAGTGAAGTTTTAAATAAAGAGTAAGATAGGAAATGAGATAAAGAGTATTCTGGAGATAGTCTTATACATCACCGTAGCAACGCATT
AATATATACCTAGTTAATATTATATGATTTAAATTTTATCTTAAAATACTTGACGCTCTATGAGTTTAGTAGTTTATAATACATATTTATCTTCTTCTAA
TACATGAACATCCTTTTTAGAATACGTCATAGGACGATCACCAGTACGGAGGATCTAGGCAAACAACATAAGAATCGAGCGAGTGCTAGGAGCATC
TCTAAAAGACTAATAAAATAACTCGTAAAGTTAAATTTTAGATACTCAAGAGTAAAATAACTATTTAACAGACTCGTTATTTGACTCATCAATATACC
CTTCAAATTGACTCTCTCACTATCTAAACTTGGCTAGCCACTATGACTAACTAAACTAGATAGATATTTTGGTGGAGTAAGATGCTATATATAAAGTG
TAATATTTATAAAATGATAAATAAAAAGCTAAAAATAAAGAGTTACTTCTCTAAGCACGCTCGTGTCCAAGCGTCACTTACGATTAGC

>ID664_HR2 junction PCR product (P5/P4), *ARGOS8-v2*
ACCGTCCTTATCTCTGCATCATCTATCTGTGCTAGTTTCTGCGTGCTTCTCAAATATTTCCGGCCTGTGTAGCATGTGACTGATAATATGATTTTGGCA
GCTTCTGCATAAGAACAACAAATCAAAAGCTTGATCAGCTCGGTGCCTACAAAACCTCAACAACCAAGTTTCCATGGTACGGATAGATATGATGCT
GCACTGCACATTGGCTATATCTGAGGCTCCTGCGCGCGCCTTGGCCAGGTGTCTGTCATGCGGGCGATGCCGCAGGAAGAGGAAGCCGCGGTGG
CGACGACGACCATGGCCGGGGGCAAGGTGGCGGCGCTGCTGGCCACGGCGGCCGCGCTGCTGCTGCTGCTCCCGCTGGCGCTGCCGCCGCTGCC
GCCGCCGCCCACGCAGCTGTTGTTCGTCCCCGTGGTCTTGCTGCTCCTCGTGGCGTCCCTCGCGTTCTGCCCCGCCGCGACCCCCTCGCCGTCGCCG
ATGCATGCCGCCGACCACGGGTCGTTCGGGACCACTGGATCACCGCACCTATGTTGACGCTGACGATGAGAGATCTCGTCGATCGACGGTCGGCT
TGCAATGGCGCGTCGGAATCAAGCAGTTCGCACAC

>ID336_PCR product (P1/P4), *ARGOS8-v3*
GCGCCATTCCCTAAAGGTAACTACTAAAACAGAACACAATTTTCTTAGGCCATCTCCAGCAGGCTGTGTAAACAGCCGTGCAAAATGTTGTTTTGCA
CTGTTTGTACAGTGAAGTTTTAAATAAAGAGTAAGATAGGAAATGAGATAAAGAGTATTCTGGAGATAGTCTTATACATCACCGTAGCAACGCATT
AATATATACCTAGTTAATATTATATGATTTAAATTTTATCTTAAAATACTTGACGCTCTATGAGTTTAGTAGTTTATAATACATATTTATCTTCTTCTAA
TACATGAACATCCTTTTTAGAATACGTCATAGGACGATCACCAGTACGGAGGATCTAGGCAAACAACATAAGAATCGAGCGAGTGCTAGGAGCATC
TCTAAAAGACTAATAAAATAACTCGTAAAGTTAAATTTTAGATACTCAAGAGTAAAATAACTATTTAACAGACTCGTTATTTGACTCATCAATATACC
CTTCAAATTGACTCTCTCACTATCTAAACTTGGCTAGCCACTATGACTAACTAAACTAGATAGATATTTTGGTGGAGTAAGATGCTATATATAAAGTG
TAATATTTATAAAATGATAAATAAAAAGCTAAAAATAAAGAGTTACTTCTCTAAGCACTCGCTGGCGCGCGGGTCCGGCGGGGCGGTCTGTGAGG
GCAAATTTATATAGGTCTAGTGGGTACCCGGCTACGGATAGATATGATGCTGCACTGCACATTGGCTATATCTGAGGCTCCTGCGCGCGCCTTGGC
CAGGTGTCTGTCATGCGGGCGATGCCGCAGGAAGAGGAAGCCGCGGTGGCGACGACGACCATGGCCGGGGGCAAGGTGGCGGCGCTGCTGGC
CACGGCGGCCGCGCTGCTGCTGCTGCTCCCGCTGGCGCTGCCGCCGCTGCCGCCGCCGCCCACGCAGCTGTTGTTCGTCCCCGTGGTCTTGCTGCT
CCTCGTGGCGTCCCTCGCGTTCTGCCCCGCCGCGACCCCCTCGCCGTCGCCGATGCATGCCGCCGACCACGGGTCGTTCGGGACCACTGGATCACC
GCACCTATGTTGACGCTGACGATGAGAGATCTCGTCGATCGACGGTCGGCTTGCAATGGCGCGTCGGAATCAAGCAGTTCGCACA

Figure S4. DNA sequences of the junction PCR products amplified from *ARGOS8* variants

Table S1. Frequency of the GOS2 promoter insertion with one target site and the promoter swap with two target sites in T0 maize plants

	Insertion	Swap
Target sites	CTS1	CTS2 and CTS3
No. of embryos bombarded	1050	1150
No. of shoots regenerated	194	334
No. of shoots with CTS modified	190	172 *
No. of shoots positive with junction PCR	7	23
HR1 junction-PCR positive	2	9
HR2 junction-PCR positive	3	11
Both junction-PCR positive	2	3
No. of T0 plants regenerated	3	8
HR1 junction-PCR positive	1	3
HR2 junction-PCR positive	1	3
Both junction-PCR positive	1	2

* Both CRISPR-RNA target site 2 (CTS2) and CTS3 were modified.

Table S2. Primers and probes used for generation and characterization of *ARGOS8* genome editing variants

Target	PCR type	Primer name	Primer type	Primer sequence (5'-3')
CTS1	qPCR	P11	Forward	CCTCTCCAACCCCAAATCCA
		P12	Reverse	CCACTAGACCTATATAAATTTGCCCTCA
			Probe	CGCCAGCGATGGAT
CTS2	qPCR	P13	Forward	TGTGAGGGCAAATTTATATAGGTCTAGTG
		P14	Reverse	AGCCTCAGATATAGCCAATGTGCA
			Probe	CCGGCTACGGATAGA
CTS3	qPCR	P15	Forward	AGATAGATATTTTGGTGGAGTAAGATGCT
		P16	Reverse	GCTAGTACTAGTAGAGGACTAGAGGCCTG
			Probe	AATCCAGCCGTGCTT
HR1 junction	PCR	P1	Forward	GCGCCATTCCCTAAAGGTAAC
		P2	Reverse	GCTAATCGTAAGTGACGCTTGGA
HR2 junction	PCR	P5	Forward	ACCGTCCTTATCTCTGCATCATCT
		P4	Reverse	GTGTGCGAACTGCTTGATTCCG
HR2 junction	PCR	P3	Forward	GCTCGTGTCCAAGCGTCACTTACGATTAGCT
		P4	Reverse	GTGTGCGAACTGCTTGATTCCG
ARGOS8 locus	Long PCR	P1	Forward	GCGCCATTCCCTAAAGGTAAC
		P4	Reverse	GTGTGCGAACTGCTTGATTCCG
Cas9	PCR	P21	Forward	AACTGGTGGACAGCACAGACAA
		P22	Reverse	GAAGTGGCCGCGGAACT
			Probe	CTCGCGCATATGAT
sgRNA	PCR	P23	Forward	CTAATCACAAGAGTGGAGCGTACCTT
		P24	Reverse	AGCCTTATTTTAACTTGCTATTTCTAGCTCT
			Probe	CCGAGCCGCAAGCA
PMI	PCR	P25	Forward	CGCCGGAGATATCGTTTCA
		P26	Reverse	CCTCTCCGAGCAGAGTCGATT
			Probe	CTCAATCACATCACGCA
ODP2	PCR	P27	Forward	TTGGTGTTACTTCTGCAGGTCGA
		P28	Reverse	AGAGGGAGAAAGCGAGCCAGT
			Probe	ATCCATGGCCACTGTGAA
WUS	PCR	P29	Forward	CTCCCTGCACCTGCCATG
		P30	Reverse	GCTGCCGCTGCCTCCTC
			Probe	CGCTGAGATAGAGGCATG

Table S3. Plant height (PLTHT), ear height (EARHT) and grain moisture (MST) of *ARGOS8* genome edited variants and wild type under flowering stress, grain-filling stress and optimal (well-watered) conditions.

	PLTHT (*cm*)	EARHT (*cm*)	MST %
		Flowering Stress (FS)	
ARGOS8-v1	213.1	94.2	20.1
ARGOS8-v2	213.1	95.0	20.4
WT	213.5	92.8	20.3
		Optimal (OPT)	
ARGOS8-v1	273.3	143.0	21.4
ARGOS8-v2	275.6*	144.5*	21.6
WT	273.0	141.3	21.5
		Grain-filling Stress (GFS)	
ARGOS8-v1	nd	nd	18.2*
ARGOS8-v2	nd	nd	18.7
WT	nd	nd	19.0

Asterisks indicate predicted difference significant at $P < 0.1$. nd, not determined.
